# Insomnia and sleep duration on COVID-19 susceptibility and hospitalization: A Mendelian randomization study

**DOI:** 10.3389/fpubh.2022.995664

**Published:** 2022-09-30

**Authors:** Liuqing Peng, Jiarui Jing, Jun Ma, Simin He, Xue Gao, Tong Wang

**Affiliations:** Department of Health Statistics and Epidemiology, School of Public Health, Shanxi Medical University, Taiyuan, China

**Keywords:** insomnia, sleep duration, COVID-19, Mendelian randomization, causal inference

## Abstract

**Background:**

Sleep disturbance including insomnia and sleep duration is associated with an increased risk of infectious. With the ongoing coronavirus disease 2019 (COVID-19) pandemic, it is important to explore potential causal associations of sleep disturbance on COVID-19 susceptibility and hospitalization.

**Method:**

Insomnia and sleep duration were selected as exposure. Outcomes included susceptibility and hospitalization for COVID-19. Two sample mendelian randomization design was used to assess causality between sleep and COVID-19. Inverse variance weighted method was used as main analysis method to combine the ratio estimates for each instrumental variable to obtain the causal effect. Cochran's Q statistic was used to test for global heterogeneity. MR-Egger and weighting median estimator (WME) were used as sensitivity analysis to ensure the stability and reliability of the results. MR-Egger intercept term was used to test the mean pleiotropy. In addition, the direct effects of insomnia and sleep duration on COVID-19 susceptibility and hospitalization were estimated using multivariable mendelian randomization (MVMR).

**Results:**

Univariate MR provided no evidence of a causal associations of insomnia on COVID-19 susceptibility (OR = 1.10, 95% CI:0.95, 1.27; *p* = 0.21) and hospitalization (OR = 0.61, 95% CI:0.40, 0.92; *p* = 0.02); as does sleep duration (OR_COIVD − 19susceptibility_ = 0.93, 95% CI:0.86, 1.01; *p* = 0.07; OR_COIVD − 19_ _hospitalization_ = 1.21, 95% CI: 0.99, 1.47; *p* = 0.08). MVMR results showed that insomnia may be a risk factor for increased susceptibility to COVID-19 (OR = 1.65, 95% CI: 1.34, 2.05; *p* <0.001); and sleep duration was also associated with increased COVID-19 susceptibility (OR = 1.31, 95% CI: 1.18, 1.46; *p* < 0.001).

**Conclusion:**

Insomnia and extreme sleep duration may risk factors for increased COVID-19 susceptibility. Relieving insomnia and maintaining normal sleep duration may be powerful measures to reduce COVID-19 infections.

## Introduction

Up to now, coronavirus disease 2019 (COVID-19) pandemic has caused more than 500 million people infected and more than 6 million people died from COVID-19 in worldwide (WHO, 2021: https://covid19.who.int). To date, there is still no specific therapy for this virus. And there are still outbreaks from time to time around the world. The isolation and treatment measures adopted in response to the COVID-19 have brought a heavy socio-economic burden to global countries. Therefore, exploring and discovering the risk factors of COVID-19, and taking appropriate protective measures for high-risk populations are important primary prevention strategies for current response to COVID-19. Sleep is an important means for relieving fatigue and improving physical function. However, sleep disturbance affect approximately one-quarter of the US population and are still increasing every year (1). Studies have shown that sleep disturbance can adversely affect the risk of infectious and inflammatory diseases (2–4). Sleep disturbances related to COVID-19 have received widespread attention, and studies from different countries have highlighted sleep quality issues in patients with COVID-19 (5–7). Insomnia and sleep duration are common complaints in sleep disturbances, and their association with COVID-19 susceptibility and hospitalization is unclear.

**Figure 1:**
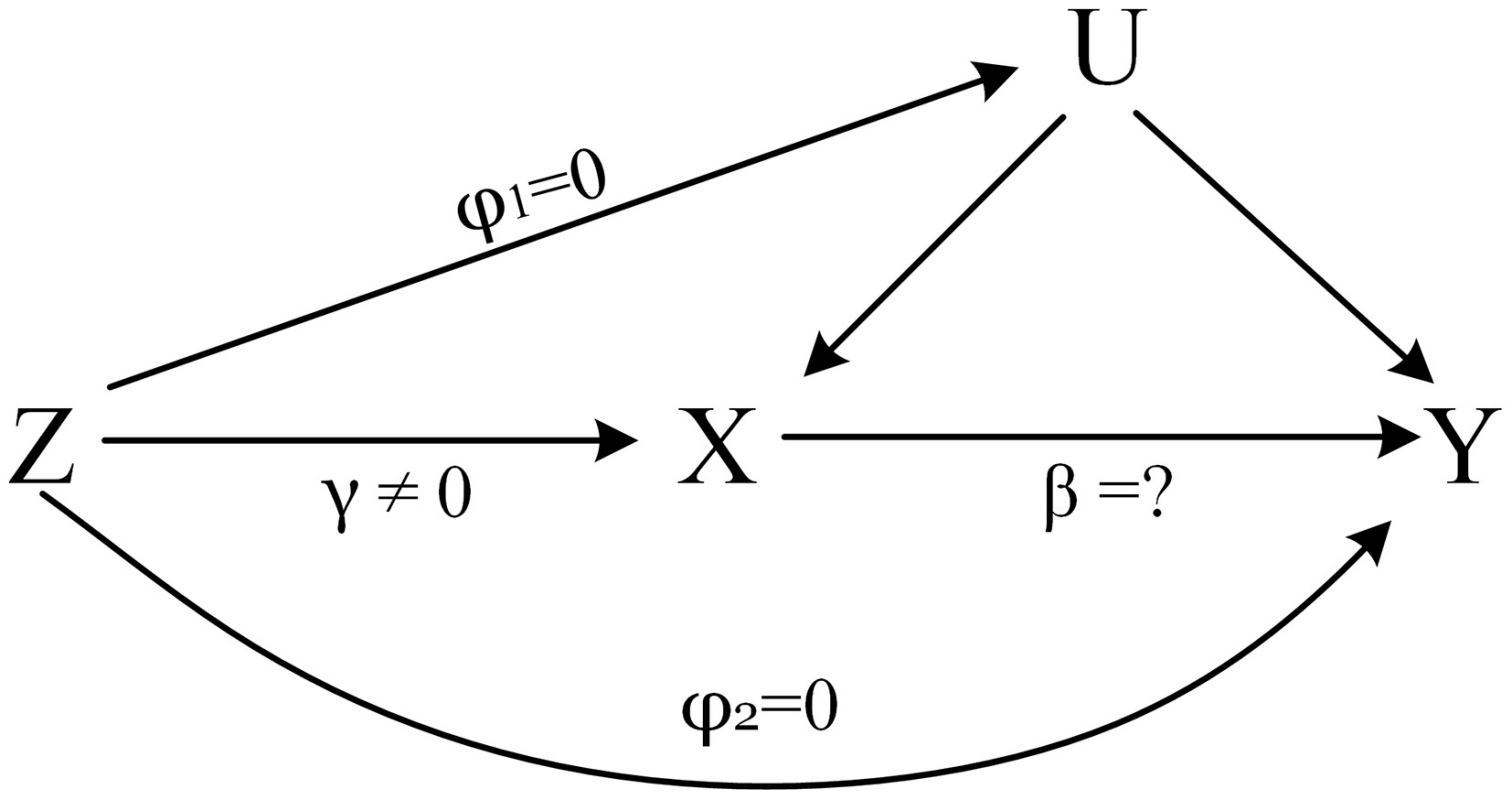
The core assumptions of MR. Z represents the genetic instrument (SNPs), X the exposure (Insomnia, Sleep duration), Y the outcome (COVID-19 Susceptibility, COVID-19 hospitalization) and U denotes the confounders of the relationship of X-Y. Assumptions: 1. Genetic variation is closely related to the exposure of interest, *γ* ≠ 0; 2. Genetic variation has nothing to do with confounding, *φ*_1_ = 0; 3. Genetic variation only affects the outcome through X, *φ*_2_ = 0.

Mendelian randomization (MR), a powerful causal inference method, uses single nucleotide polymorphisms (SNPs) as instrumental variables to estimate the causal effect of an exposure on an outcome (8, 9). It is less affected by confounding and reverse causality than traditional observational studies (10). The core assumptions of MR were shown in Figure 1 (11).

This study used univariate MR to explore the potential causal relationship of insomnia, sleep duration on COVID-19 susceptibility and hospitalization, and multivariable Mendelian randomization (MVMR) (12) was used to analyze the effects of insomnia and sleep duration on COVID-19 susceptibility and hospitalization.

## Materials and methods

### Data sources

In this study, insomnia and sleep duration were selected as exposures. COVID-19 susceptibility and COVID-19 hospitalization were outcomes. Summary-level data on sleep and COVID-19 were obtained from four published genome wide association studies (GWAS). And ethical approval had been obtained for these GWAS (Table 1).

**Table 1:** GWAS cohorts used in this study.

**Phenotype**	**First author (year)**	**Sample size**	**Consortium**
Insomnia	Jacqueline M. Lane (2019)	453,379	UK Biobank
Sleep duration	Hassan S. Dashti (2019)	446,118	UK Biobank
COVID-19 susceptibility		32,494 cases, 1,316,207 controls	COVID-19 host genetics initiative
COVID-19 hospitalization		8,316 cases, 1,549,095 controls	COVID-19 host genetics initiative

Summary-level data for insomnia were derived from the largest available meta-analysis of GWAS, including unrelated European descent individuals from UK Biobank (UKB, *N* = 453,379, cases = 345,022, control = 108,357). Self-reported insomnia symptoms were determined by answering questions such as “Do you have trouble falling asleep at night?”, “do you wake up in the middle of the night?” (13).

Genetic association estimates with sleep duration were obtained from the UKB participants of European ancestry (*N* = 446,118, 55.9% male) (14). The mean self-reported sleep duration was 7.2 h (standard deviation = 1.1 h). Sleep duration was treated as a continuous variable, and assessed by the question: “How many hours do you sleep per day (including naps)?” The answer was responded in hour increments and could only contain integer values.

GWAS for COVID-19 susceptibility and hospitalization were obtained from the COVID-19 Host Genetics Initiative (15). COVID-19 susceptibility was a group of laboratory-confirmed or self-reported COVID-19 cases (*N* = 32,494) compared to the rest of the population (including all individuals who were not part of the case group, *N* = 1,316,207). COVID-19 hospitalization was measured based on the group hospitalized for COVID-19 (*N* = 8,316) vs. the rest of the population (*N* = 1,549,095). The participants were all of European ancestry, and details are available from the COVID-19 Host Genetics Program homepage (https://www.covid19hg.org/).

As the exposure samples were from the UK Biobank, we excluded subjects from the UK Biobank when selecting outcome samples to avoid bias due to sample overlap in MR analysis.

### Selection of SNPs

To avoid potential weak instrumental bias (16), we used genome-wide significance (*p* < 5 × 10^−8^) as the threshold for inclusion of SNPs. The instrumental variable strength was assessed using F statistic (17). To further ensure instruments independence, SNPs in linkage disequilibrium (LD) were removed from the instrument variable set (r^2^ threshold = 0.001, window size = 10,000 kb). The F-statistics corresponding to the SNPs finally included in the MR model were all above 30, indicating that weak instrumental variable bias was unlikely (more details seen in Supplementary material 1).

### Main MR analysis

In univariate MR, Inverse variance weight (IVW) (18) was used as the main analysis method, and global Q test (19) was used to detect the presence of heterogeneity among the effect estimates from individual SNPs. If there was heterogeneity, the random effect IVW model was selected; otherwise, the IVW method of the fixed effect model was selected. In MVMR, insomnia and sleep duration were used as exposures simultaneously to explore their effects on COVID-19 susceptibility and hospitalization.

### Sensitivity analysis

MR-Egger (20) and weighting median estimator (WME) (21) were used as sensitivity analysis methods. MR-Egger provides a valid test for pleiotropy, and the estimates of the intercept term in MR-Egger can be interpreted as the average pleiotropic effects across SNPs. And the estimator of WME is consistent even when up to 50% of the information comes from invalid SNPs. In addition, to test the reverse causality, we also performed reverse MR analysis.

To account for multiple testing, we employed a Bonferroni-corrected threshold of *p* < 0.0125 (0.05/4 to correct for two sleep traits in relation to COVID-19 susceptibility and hospitalization). *P* values between 0.0125 and 0.05 were considered suggestive evidence of causality, requiring confirmation. All statistical analyses were performed using R (version 4.2.0.) software, and univariate MR and MVMR analyses were performed using the TwosampleMR and Mendelian Randomization packages, respectively.

## Results

### Results of univariable Mendelian randomization

IVW showed that the effect of insomnia on COVID-19 susceptibility was not statistically significant (OR = 1.10, 95% CI: 0.95, 1.27; *p* = 0.21), the sensitivity analysis method results were consistent with IVW method. In the analysis of the impact of insomnia on COVID-19 hospitalization, IVW method and the sensitivity analysis results showed a suggestive causal association between insomnia and COVID-19 hospitalization (OR = 0.61, 95% CI: 0.40, 0.92; *p* = 0.02) (Table 2). There was no causal association of sleep duration on COVID-19 susceptibility (OR = 0.93, 95% CI: 0.86, 1.01; *p* = 0.07). Sleep duration also had no causal effect on COVID-19 hospitalization (OR = 1.21, 95% CI: 0.99, 1.47; *p* = 0.08), the results of the sensitivity analysis methods were consistent with IVW method (Table 2).

**Table 2:** The effect of insomnia, sleep duration on COVID-19.

**Exposure**	**Outcome**	**Methods**	**nSNPs**	**OR (95% CI)**	***P*** **-value**
Insomnia	COVID-19 susceptibility	IVW	40	1.10 (0.95,1.27)	0.21
		MR-Egger	40	1.10 (0.66,1.84)	0.72
		Egger-Intercept		−1.38 × 10^−5^	0.99
		WME	40	1.05 (0.85,1.29)	0.65
Insomnia	COVID-19 hospitalization	IVW	40	0.61 (0.40,0.92)	0.02
		MR-Egger	40	1.98 (0.41,9.58)	0.40
		Egger-Intercept		−0.01	0.13
		WME	40	0.54 (0.31,0.95)	0.03
Sleep duration	COVID-19 susceptibility	IVW	67	0.93 (0.86,1.01)	0.07
		MR-Egger	67	0.80 (0.59,1.07)	0.26
		Egger-Intercept		0.0027	0.29
		WME	67	0.94 (0.85,1.05)	0.42
Sleep duration	COVID-19 hospitalization	IVW	67	1.21 (0.99,1.47)	0.08
		MR-Egger	67	1.56 (0.72,3.36)	0.14
		Egger-Intercept		−0.0044	0.50
		WME	67	1.12 (0.85,1.49)	0.08

**Figure 2:**
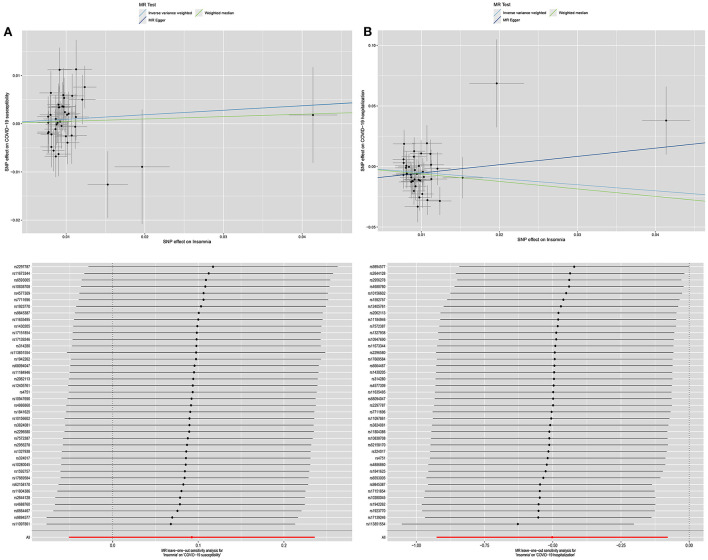
The scatterplot and leave-one-out results of the univariate MR analysis. **(A)** is the scatterplot and leave-one-out results of the effect of insomnia on COVID-19 susceptibility in univariate MR analysis. **(B)** is the scatterplot and leave-one-out results of the effect of insomnia on COVID-19 hospitalization in univariate MR analysis.

The scatterplot and leave-one-out results of the univariate MR analysis were shown in Figure 2.

### Results of MVMR

Insomnia and sleep duration were used as exposures to estimate their effects on COVID-19 susceptibility and hospitalization. MVMR results suggested that insomnia may be a risk factor for COVID-19 susceptibility (OR = 1.65, 95% CI: 1.34, 2.05; *p* < 0.001); Sleep duration also linked to increased COVID-19 susceptibility (OR = 1.31, 95% CI: 1.18, 1.46; *p* < 0.001). However, we did not find evidence of potential causal relationship of insomnia and sleep duration on COVID-19 hospitalization (OR_COIVD − 19 susceptibility_ = 0.74, 95% CI: 0.42, 1.30, *p* = 0.30; OR_COVID − 19 hospitalization_=1.04, 95% CI: 0.78, 1.40, *p* = 0.77) (Table 3).

**Table 3:** The results of multivariable MR.

**Exposure**	**Outcome**	**nSNPs**	**OR (95% CI)**	**P-value**
Insomnia	COVID-19 susceptibility	383	1.65 (1.34,2.05)	<0.001
Sleep duration		383	1.31 (1.18,1.46)	<0.001
Insomnia	COVID-19 hospitalization	380	0.74 (0.42,1.30)	0.30
Sleep duration		380	1.04 (0.78,1.40)	0.77

## Discussion

We explored potential causal associations between insomnia and sleep duration on COVID-19 susceptibility and hospitalization using two sample MR. Univariate MR showed no causal associations of insomnia and sleep duration with COVID-19 susceptibility and hospitalization. MVMR showed that both insomnia and sleep duration were risk factors for increased COVID-19 susceptibility, but there was no causal relationship with COVID-19 hospitalization. We found that univariate MR and MVMR results contradicted, and the reason for this may be that in the insomnia GWAS cohort used in univariate MR, the sleep duration of insomnia patients changed due to insomnia, that means, the sample had selection bias (22, 23). For this reason, the assumption 2 of MR may be violated (Figure 1), thereby the results of univariate MR can be biased. The reverse MR results also further verified this conjecture, reverse MR results showed a causal association between COVID-19 susceptibility and sleep duration (Supplementary Table 1), while insomnia had an impact on sleep duration (Supplementary Table 2). This suggested that sleep duration is a potential collider factor in univariate MR analysis (22), which lead to substantially biased estimates of associations in univariate MR. Notably, research showed that MR results are susceptible to selection bias, and when it is exists, the closer the true estimate is to the null, the easier it is for a reversal to occur (24). While MVMR is not affected by selection bias and collider factors, direct effects of different exposures on outcomes can be obtained (24, 25). Above all, the results of MVMR are more reliable, there is reason to believe that insomnia and sleep duration are risk factors for increased COVID-19 susceptibility. What's more, there was growing evidence that sleep disturbance can adversely affect adaptive immunity in humans. Sleep disturbance can activate the hypothalamic-pituitary-adrenal axis, increase the release of glucocorticoid, and weaken the body's immune response through the following three pathways. First, activation of the glucocorticoid receptor inhibits the transcription of many immune response genes; second, activation of the glucocorticoid receptor induces the transcription of certain anti-inflammatory genes (such as NFKBIA, which encodes IκBα), which interferes with the activation of the transcription factor, resulting in the blockade of the inflammatory cascade; finally, protein-protein interactions interfere with the pro-inflammatory transcription factors NF-κB and AP-1, antagonizing the transcription of inflammatory genes (26). Insomnia independently increases the risk of infectious and inflammatory diseases (27, 28), and Irwin et al. demonstrated that patients with insomnia exhibited a reduction in natural killer cells, which may lead to a decline in the body's natural immunity (29). Furthermore, extreme sleep duration is associated with infectious disease risk, and epidemiological evidence suggested that reduced (e.g., ≤5 hours) and prolonged (e.g., ≥ 9 h) sleep duration was associated with an increased risk of pneumonia (30). The above mechanisms may be the reasons for the increased susceptibility of COVID-19 in patients with insomnia. The sleep disturbance may be related to the confinement, anxiety and other psychosocial factors brought about by COVID-19, rather than COVID-19 itself (31).

Therefore, we suggested that in the future prevention and control of COVID-19, attention should be paid to people with sleep disorders, and taking necessary protective measures will help reduce their COVID-19 susceptibility. Insomnia is a modifiable risk factor, and studies have shown that some relaxation therapies, such as tai chi and yoga, can greatly improve symptoms of insomnia (32, 33). To sum up, carrying out necessary relaxation training in people with insomnia may reduce the risk of COVID-19 infection, alleviate the potential pressure and economic expenditure of COVID-19 epidemic prevention and control, and is expected to become an important primary preventive measure.

### Strength and limitation

This study used two sample MR to explore the causal association between insomnia and sleep duration on COVID-19 susceptibility and hospitalization, compared with traditional observational studies, it is less susceptible to reverse causality and confounding. Furthermore, we found that due to the close biological association between sleep traits, univariate MR is susceptible to selection bias and it is difficult to draw correct conclusions. We suggested when performing univariate MR, it is necessary to fully consider the impact of other traits that are closely related to the exposure of interest on study results, and whether this will cause the violation of the core assumptions of MR. This study assumed a linear association between the exposures of interest and the outcomes, and the nonlinear association still needs to be further explored.

## Conclusion

Insomnia and sleep duration are risk factors for increased COVID-19 susceptibility. Relieving insomnia and maintaining normal sleep duration may be a powerful measure to reduce COVID-19 infection.

## Data availability statement

Summary level data of this article are publicly available, sleep traits can be found at https://sleep.hugeamp.org/dinspector.html?dataset=GWAS_UKBB_eu. COVID-19 susceptibility and hospitalization can be found at https://www.covid19hg.org/.

## Author contributions

TW, LP, and JJ designed the whole study. JM acquired and interpreted the data. LP and JJ performed the statistical analysis and drafted the manuscript. All authors read and approved the final manuscript.

## Funding

This study was supported by the National Natural Science Foundation of China [Grant Numbers 81872715, 82073674, and 82103949], the Basic Research Project of Shanxi Province, China [Grant Number 20210302124186], and the Major Science and Technology Project of Shanxi Province [Grant Numbers 202102130501003 and 202005D121008].

## Conflict of interest

The authors declare that the research was conducted in the absence of any commercial or financial relationships that could be construed as a potential conflict of interest.

## Publisher's note

All claims expressed in this article are solely those of the authors and do not necessarily represent those of their affiliated organizations, or those of the publisher, the editors and the reviewers. Any product that may be evaluated in this article, or claim that may be made by its manufacturer, is not guaranteed or endorsed by the publisher.
