# Supplementary material for: Insomnia and sleep duration on COVID-19 susceptibility and hospitalization: A Mendelian randomization study
Source: Front Public Health. 2022 Sep 30;10:995664. doi: 10.3389/fpubh.2022.995664 (PMC9561394; doi:10.3389/fpubh.2022.995664)
Supplement: Supplementary file 1 [file Data_Sheet_1.docx]

Table 1. SNPs used for univariable MR analysis estimating the effect of COVID-19 hospitalization on insomnia, EA=effect_allele, OA=other_allele

|  | SNP | EA | OA | beta.exposure | beta.outcome | se.outcome | se.exposure | F value |
| --- | --- | --- | --- | --- | --- | --- | --- | --- |
| 1 | rs10774679 | C | T | 0.075824 | -0.00316 | 0.001434 | 0.010966 | 47.8098 |
| 2 | rs111837807 | T | C | 0.11295 | -0.00773 | 0.002815 | 0.018461 | 37.43364 |
| 3 | rs11702230 | C | T | 0.099731 | -0.00161 | 0.002154 | 0.016847 | 35.04412 |
| 4 | rs12809318 | T | C | -0.06278 | 0.003064 | 0.001381 | 0.01094 | 32.93228 |
| 5 | rs13050728 | T | C | -0.10657 | -0.00134 | 0.0015 | 0.0113 | 88.94326 |
| 6 | rs1405655 | T | C | 0.075181 | 0.001526 | 0.001473 | 0.011072 | 46.1067 |
| 7 | rs2277732 | C | A | 0.11697 | 0.002232 | 0.001494 | 0.012151 | 92.66696 |
| 8 | rs34536443 | G | C | 0.17443 | 0.001127 | 0.003414 | 0.030989 | 31.68307 |
| 9 | rs35508621 | T | C | 0.38931 | 0.000967 | 0.00273 | 0.019471 | 399.7741 |
| 10 | rs35705950 | G | T | -0.11177 | 0.002267 | 0.002188 | 0.019621 | 32.44952 |
| 11 | rs505922 | C | T | -0.09988 | 6.42E-05 | 0.001487 | 0.010789 | 85.7096 |
| 12 | rs61667602 | T | C | -0.09355 | 0.002715 | 0.00166 | 0.013718 | 46.50966 |
| 13 | rs67579710 | G | A | -0.10348 | -4.73E-05 | 0.002238 | 0.01695 | 37.27121 |
| 14 | rs766826 | C | T | -0.08213 | 0.001223 | 0.001473 | 0.012104 | 46.0456 |

Table 2. SNPs used for univariable MR analysis estimating the effect of COVID-19 hospitalization on sleep duration, EA=effect_allele, OA=other_allele

|  | SNP | EA | OA | beta.exposure | beta.outcome | se.outcome | se.exposure | F value |
| --- | --- | --- | --- | --- | --- | --- | --- | --- |
| 1 | rs10774679 | C | T | 0.075824 | 0.001252 | 0.002351 | 0.010966 | 47.8098 |
| 2 | rs111837807 | T | C | 0.11295 | 0.0092 | 0.004611 | 0.018461 | 37.43364 |
| 3 | rs11702230 | C | T | 0.099731 | 0.004665 | 0.003521 | 0.016847 | 35.04412 |
| 4 | rs12809318 | T | C | -0.06278 | -0.00035 | 0.002262 | 0.01094 | 32.93228 |
| 5 | rs13050728 | T | C | -0.10657 | -0.00102 | 0.00245 | 0.0113 | 88.94326 |
| 6 | rs1405655 | T | C | 0.075181 | 0.001329 | 0.002413 | 0.011072 | 46.1067 |
| 7 | rs2277732 | C | A | 0.11697 | -0.00096 | 0.002442 | 0.012151 | 92.66696 |
| 8 | rs34536443 | G | C | 0.17443 | 0.007311 | 0.00558 | 0.030989 | 31.68307 |
| 9 | rs35508621 | T | C | 0.38931 | 0.005559 | 0.004466 | 0.019471 | 399.7741 |
| 10 | rs35705950 | G | T | -0.11177 | -0.00714 | 0.003588 | 0.019621 | 32.44952 |
| 11 | rs505922 | C | T | -0.09988 | -0.00874 | 0.002436 | 0.010789 | 85.7096 |
| 12 | rs61667602 | T | C | -0.09355 | 0.015678 | 0.002711 | 0.013718 | 46.50966 |
| 13 | rs67579710 | G | A | -0.10348 | 0.001632 | 0.003667 | 0.01695 | 37.27121 |
| 14 | rs766826 | C | T | -0.08213 | -0.00229 | 0.00241 | 0.012104 | 46.0456 |

Table 3. SNPs used for univariable MR analysis estimating the effect of insomnia on COVID-19 hospitalization, EA=effect_allele, OA=other_allele

|  | SNP | EA | OA | beta.exposure | beta.outcome | se.outcome | se.exposure | F value |
| --- | --- | --- | --- | --- | --- | --- | --- | --- |
| 1 | rs10156602 | A | G | 0.010112 | -0.02271 | 0.011578 | 0.001448 | 48.75736 |
| 2 | rs10280045 | C | G | -0.00907 | -0.01261 | 0.011461 | 0.001408 | 41.46565 |
| 3 | rs10838708 | G | A | 0.008402 | -0.00031 | 0.010946 | 0.0014 | 36.00831 |
| 4 | rs10947690 | A | G | -0.00869 | 0.012746 | 0.013694 | 0.001574 | 30.46427 |
| 5 | rs11097861 | A | G | -0.00916 | 0.002953 | 0.012594 | 0.001535 | 35.64079 |
| 6 | rs11184946 | C | T | -0.00879 | 0.012188 | 0.010924 | 0.0014 | 39.40735 |
| 7 | rs113851554 | G | T | -0.04137 | -0.03791 | 0.028066 | 0.003074 | 181.1584 |
| 8 | rs11635495 | T | C | -0.00813 | 0.00556 | 0.011081 | 0.001386 | 34.42501 |
| 9 | rs11673344 | A | G | -0.00869 | 0.00894 | 0.011509 | 0.001429 | 36.94487 |
| 10 | rs11804386 | G | A | -0.00806 | -0.0006 | 0.011751 | 0.001464 | 30.28288 |
| 11 | rs12405761 | A | C | 0.009239 | -0.01637 | 0.011409 | 0.001399 | 43.64463 |
| 12 | rs1327938 | T | C | -0.00912 | 0.010668 | 0.01197 | 0.001467 | 38.65856 |
| 13 | rs1430205 | C | T | -0.00769 | 0.006994 | 0.011285 | 0.001391 | 30.5964 |
| 14 | rs1592757 | G | C | -0.00904 | 0.020195 | 0.011517 | 0.001444 | 39.19375 |
| 15 | rs17139246 | T | C | -0.00778 | -0.0187 | 0.01147 | 0.001429 | 29.59429 |
| 16 | rs17151854 | G | T | -0.01071 | -0.01917 | 0.014866 | 0.001923 | 30.99403 |
| 17 | rs17669584 | A | G | -0.00965 | 0.011025 | 0.015552 | 0.001784 | 29.24091 |
| 18 | rs1841625 | A | G | -0.00771 | -0.00587 | 0.010932 | 0.001398 | 30.40864 |
| 19 | rs1923770 | T | A | 0.009963 | 0.010774 | 0.011022 | 0.001424 | 48.96471 |
| 20 | rs1942262 | G | A | -0.01115 | -0.01043 | 0.01179 | 0.001523 | 53.61431 |
| 21 | rs2062113 | T | C | 0.0089 | -0.01207 | 0.010636 | 0.001403 | 40.22037 |
| 22 | rs2296580 | G | T | 0.010313 | -0.00862 | 0.012014 | 0.00151 | 46.62437 |
| 23 | rs2297787 | T | A | 0.015286 | -0.00928 | 0.017023 | 0.002561 | 35.61741 |
| 24 | rs2644128 | C | G | -0.00974 | 0.025554 | 0.010882 | 0.001387 | 49.28142 |
| 25 | rs2956278 | A | G | -0.00952 | 0.033134 | 0.012859 | 0.001685 | 31.9532 |
| 26 | rs314280 | A | G | -0.00864 | 0.006777 | 0.010997 | 0.001389 | 38.66559 |
| 27 | rs324017 | A | C | 0.009676 | 0.00048 | 0.012044 | 0.001519 | 40.57706 |
| 28 | rs3824081 | T | C | 0.008014 | -0.00113 | 0.010991 | 0.001389 | 33.27518 |
| 29 | rs4577309 | A | G | 0.008118 | -0.00645 | 0.011628 | 0.001388 | 34.19094 |
| 30 | rs4688760 | C | T | -0.01077 | 0.027795 | 0.012268 | 0.001499 | 51.56463 |
| 31 | rs4751 | G | T | -0.00773 | -0.00301 | 0.011087 | 0.001397 | 30.61285 |
| 32 | rs4886860 | G | C | 0.011237 | 0.001437 | 0.012494 | 0.001633 | 47.36362 |
| 33 | rs62158170 | A | G | 0.012052 | -0.00178 | 0.013606 | 0.001683 | 51.28256 |
| 34 | rs6593005 | A | G | -0.00903 | -0.00807 | 0.011609 | 0.00158 | 32.67987 |
| 35 | rs6664467 | G | A | 0.011265 | -0.01039 | 0.015732 | 0.002016 | 31.23964 |
| 36 | rs68094047 | C | T | -0.00942 | 0.006116 | 0.01206 | 0.0016 | 34.62695 |
| 37 | rs7572387 | A | C | -0.00984 | 0.011669 | 0.011237 | 0.001402 | 49.21776 |
| 38 | rs7711696 | G | T | -0.01018 | 0.00441 | 0.01126 | 0.001501 | 45.97555 |
| 39 | rs9845387 | C | A | 0.019653 | 0.068612 | 0.036294 | 0.003501 | 31.50628 |
| 40 | rs9894577 | G | A | -0.01236 | 0.028458 | 0.01156 | 0.001487 | 69.09064 |

Table 4. SNPs used for univariable MR analysis estimating the effect of insomnia on sleep duration, EA=effect_allele, OA=other_allele

|  | SNP | EA | OA | beta.exposure | beta.outcome | se.outcome | se.exposure | F value |
| --- | --- | --- | --- | --- | --- | --- | --- | --- |
| 1 | rs10156602 | A | G | 0.010112 | -0.00235 | 0.002373 | 0.001448 | 48.75736 |
| 2 | rs10280045 | C | G | -0.00907 | 0.015266 | 0.002304 | 0.001408 | 41.46565 |
| 3 | rs10838708 | G | A | 0.008402 | -0.00815 | 0.00229 | 0.0014 | 36.00831 |
| 4 | rs10947690 | A | G | -0.00869 | 0.004345 | 0.002576 | 0.001574 | 30.46427 |
| 5 | rs11097861 | A | G | -0.00916 | 0.009294 | 0.002512 | 0.001535 | 35.64079 |
| 6 | rs11184946 | C | T | -0.00879 | 0.005066 | 0.002294 | 0.0014 | 39.40735 |
| 7 | rs113851554 | G | T | -0.04137 | 0.013957 | 0.00505 | 0.003074 | 181.1584 |
| 8 | rs11635495 | T | C | -0.00813 | -0.00105 | 0.002267 | 0.001386 | 34.42501 |
| 9 | rs11673344 | A | G | -0.00869 | 0.003086 | 0.002337 | 0.001429 | 36.94487 |
| 10 | rs11804386 | G | A | -0.00806 | 0.00347 | 0.002397 | 0.001464 | 30.28288 |
| 11 | rs12405761 | A | C | 0.009239 | -0.01177 | 0.002287 | 0.001399 | 43.64463 |
| 12 | rs1327938 | T | C | -0.00912 | 0.002872 | 0.002402 | 0.001467 | 38.65856 |
| 13 | rs1430205 | C | T | -0.00769 | 0.007262 | 0.002275 | 0.001391 | 30.5964 |
| 14 | rs1592757 | G | C | -0.00904 | 0.000522 | 0.002362 | 0.001444 | 39.19375 |
| 15 | rs17139246 | T | C | -0.00778 | -0.00296 | 0.002342 | 0.001429 | 29.59429 |
| 16 | rs17151854 | G | T | -0.01071 | 0.002679 | 0.003155 | 0.001923 | 30.99403 |
| 17 | rs17669584 | A | G | -0.00965 | 0.006747 | 0.002913 | 0.001784 | 29.24091 |
| 18 | rs1841625 | A | G | -0.00771 | 0.000385 | 0.002286 | 0.001398 | 30.40864 |
| 19 | rs1923770 | T | A | 0.009963 | -0.0102 | 0.002332 | 0.001424 | 48.96471 |
| 20 | rs1942262 | G | A | -0.01115 | 0.010648 | 0.00249 | 0.001523 | 53.61431 |
| 21 | rs2062113 | T | C | 0.0089 | -0.00267 | 0.002294 | 0.001403 | 40.22037 |
| 22 | rs2296580 | G | T | 0.010313 | -0.00607 | 0.002471 | 0.00151 | 46.62437 |
| 23 | rs2297787 | T | A | 0.015286 | 0.01001 | 0.004211 | 0.002561 | 35.61741 |
| 24 | rs2644128 | C | G | -0.00974 | 0.010723 | 0.002274 | 0.001387 | 49.28142 |
| 25 | rs2956278 | A | G | -0.00952 | 0.004013 | 0.002758 | 0.001685 | 31.9532 |
| 26 | rs314280 | A | G | -0.00864 | 0.007163 | 0.002273 | 0.001389 | 38.66559 |
| 27 | rs324017 | A | C | 0.009676 | -0.01125 | 0.002486 | 0.001519 | 40.57706 |
| 28 | rs3824081 | T | C | 0.008014 | -0.00591 | 0.002271 | 0.001389 | 33.27518 |
| 29 | rs4577309 | A | G | 0.008118 | -0.00828 | 0.002274 | 0.001388 | 34.19094 |
| 30 | rs4688760 | C | T | -0.01077 | 0.008463 | 0.002451 | 0.001499 | 51.56463 |
| 31 | rs4751 | G | T | -0.00773 | 0.004455 | 0.002288 | 0.001397 | 30.61285 |
| 32 | rs4886860 | G | C | 0.011237 | -0.01182 | 0.002672 | 0.001633 | 47.36362 |
| 33 | rs575346808 | T | A | -0.01052 | 0.000298 | 0.002558 | 0.001562 | 45.30137 |
| 34 | rs62158170 | A | G | 0.012052 | -0.04073 | 0.002763 | 0.001683 | 51.28256 |
| 35 | rs6593005 | A | G | -0.00903 | 0.003252 | 0.002585 | 0.00158 | 32.67987 |
| 36 | rs6664467 | G | A | 0.011265 | -0.01153 | 0.003301 | 0.002016 | 31.23964 |
| 37 | rs68094047 | C | T | -0.00942 | 0.012934 | 0.00262 | 0.0016 | 34.62695 |
| 38 | rs7572387 | A | C | -0.00984 | 0.010746 | 0.002295 | 0.001402 | 49.21776 |
| 39 | rs7711696 | G | T | -0.01018 | 0.012783 | 0.002458 | 0.001501 | 45.97555 |
| 40 | rs9845387 | C | A | 0.019653 | -0.01104 | 0.005729 | 0.003501 | 31.50628 |
| 41 | rs9894577 | G | A | -0.01236 | 0.004711 | 0.002435 | 0.001487 | 69.09064 |

Table 5. SNPs used for univariable MR analysis estimating the effect of insomnia on COVID-19 susceptibility, EA=effect_allele, OA=other_allele

|  | SNP | EA | OA | beta.exposure | beta.outcome | se.outcome | se.exposure | F value |
| --- | --- | --- | --- | --- | --- | --- | --- | --- |
| 1 | rs10156602 | A | G | 0.010112 | 0.001856 | 0.004335 | 0.001448 | 48.75736 |
| 2 | rs10280045 | C | G | -0.00907 | -0.00339 | 0.004218 | 0.001408 | 41.46565 |
| 3 | rs10838708 | G | A | 0.008402 | -0.00558 | 0.004311 | 0.0014 | 36.00831 |
| 4 | rs10947690 | A | G | -0.00869 | -0.00099 | 0.00499 | 0.001574 | 30.46427 |
| 5 | rs11097861 | A | G | -0.00916 | -0.0112 | 0.004608 | 0.001535 | 35.64079 |
| 6 | rs11184946 | C | T | -0.00879 | 0.000152 | 0.00425 | 0.0014 | 39.40735 |
| 7 | rs113851554 | G | T | -0.04137 | -0.00181 | 0.009929 | 0.003074 | 181.1584 |
| 8 | rs11635495 | T | C | -0.00813 | 0.002215 | 0.004185 | 0.001386 | 34.42501 |
| 9 | rs11673344 | A | G | -0.00869 | 0.006788 | 0.004288 | 0.001429 | 36.94487 |
| 10 | rs11804386 | G | A | -0.00806 | -0.00641 | 0.004527 | 0.001464 | 30.28288 |
| 11 | rs12405761 | A | C | 0.009239 | 0.000459 | 0.004205 | 0.001399 | 43.64463 |
| 12 | rs1327938 | T | C | -0.00912 | -0.00343 | 0.004457 | 0.001467 | 38.65856 |
| 13 | rs1430205 | C | T | -0.00769 | 0.001977 | 0.0042 | 0.001391 | 30.5964 |
| 14 | rs1592757 | G | C | -0.00904 | -0.00396 | 0.004372 | 0.001444 | 39.19375 |
| 15 | rs17139246 | T | C | -0.00778 | 0.001721 | 0.004325 | 0.001429 | 29.59429 |
| 16 | rs17151854 | G | T | -0.01071 | 0.002429 | 0.005877 | 0.001923 | 30.99403 |
| 17 | rs17669584 | A | G | -0.00965 | -0.00594 | 0.005604 | 0.001784 | 29.24091 |
| 18 | rs1841625 | A | G | -0.00771 | -0.00147 | 0.004182 | 0.001398 | 30.40864 |
| 19 | rs1923770 | T | A | 0.009963 | -0.00261 | 0.004258 | 0.001424 | 48.96471 |
| 20 | rs1942262 | G | A | -0.01115 | 0.000624 | 0.00457 | 0.001523 | 53.61431 |
| 21 | rs2062113 | T | C | 0.0089 | 0.000219 | 0.004189 | 0.001403 | 40.22037 |
| 22 | rs2296580 | G | T | 0.010313 | 0.002081 | 0.004577 | 0.00151 | 46.62437 |
| 23 | rs2297787 | T | A | 0.015286 | -0.01262 | 0.00691 | 0.002561 | 35.61741 |
| 24 | rs2644128 | C | G | -0.00974 | -0.00535 | 0.004173 | 0.001387 | 49.28142 |
| 25 | rs2956278 | A | G | -0.00952 | -0.00359 | 0.005026 | 0.001685 | 31.9532 |
| 26 | rs314280 | A | G | -0.00864 | 0.00112 | 0.004149 | 0.001389 | 38.66559 |
| 27 | rs324017 | A | C | 0.009676 | 0.003534 | 0.004516 | 0.001519 | 40.57706 |
| 28 | rs3824081 | T | C | 0.008014 | 0.00185 | 0.004172 | 0.001389 | 33.27518 |
| 29 | rs4577309 | A | G | 0.008118 | -0.00481 | 0.004243 | 0.001388 | 34.19094 |
| 30 | rs4688760 | C | T | -0.01077 | -0.00579 | 0.004526 | 0.001499 | 51.56463 |
| 31 | rs4751 | G | T | -0.00773 | -0.00024 | 0.004465 | 0.001397 | 30.61285 |
| 32 | rs4886860 | G | C | 0.011237 | 0.001404 | 0.004838 | 0.001633 | 47.36362 |
| 33 | rs62158170 | A | G | 0.012052 | 0.005007 | 0.00511 | 0.001683 | 51.28256 |
| 34 | rs6593005 | A | G | -0.00903 | 0.006289 | 0.004622 | 0.00158 | 32.67987 |
| 35 | rs6664467 | G | A | 0.011265 | 0.011294 | 0.006128 | 0.002016 | 31.23964 |
| 36 | rs68094047 | C | T | -0.00942 | 0.000474 | 0.004706 | 0.0016 | 34.62695 |
| 37 | rs7572387 | A | C | -0.00984 | -0.00241 | 0.004179 | 0.001402 | 49.21776 |
| 38 | rs7711696 | G | T | -0.01018 | 0.003924 | 0.004477 | 0.001501 | 45.97555 |
| 39 | rs9845387 | C | A | 0.019653 | -0.00894 | 0.011888 | 0.003501 | 31.50628 |
| 40 | rs9894577 | G | A | -0.01236 | -0.00761 | 0.004424 | 0.001487 | 69.09064 |

Table 6. SNPs used for univariable MR analysis estimating the effect of sleep duration on COVID-19 hospitalization, EA=effect_allele, OA=other_allele

|  | SNP | EA | OA | beta.exposure | beta.outcome | se.outcome | se.exposure | F value |
| --- | --- | --- | --- | --- | --- | --- | --- | --- |
| 1 | rs10173260 | T | C | -0.01284 | 0.003135 | 0.011101 | 0.002313 | 30.79496 |
| 2 | rs10421649 | T | A | -0.0133 | 0.03694 | 0.011903 | 0.002295 | 33.58292 |
| 3 | rs10483350 | A | G | -0.01737 | 0.009906 | 0.014464 | 0.002868 | 36.68702 |
| 4 | rs10761674 | C | T | 0.012333 | -0.00632 | 0.010418 | 0.002266 | 29.6241 |
| 5 | rs1079727 | T | C | -0.01829 | 0.004223 | 0.014389 | 0.003103 | 34.74056 |
| 6 | rs10973207 | G | T | -0.02043 | -0.02102 | 0.015656 | 0.003124 | 42.78555 |
| 7 | rs11039544 | G | A | 0.018389 | -0.00868 | 0.013748 | 0.003074 | 35.7947 |
| 8 | rs11190970 | G | A | 0.015379 | 0.004846 | 0.014109 | 0.002823 | 29.6742 |
| 9 | rs112230981 | A | G | 0.031528 | 0.053478 | 0.031123 | 0.005228 | 36.36885 |
| 10 | rs113113059 | T | C | 0.016141 | 0.038134 | 0.012749 | 0.002737 | 34.76868 |
| 11 | rs11567976 | C | T | -0.0128 | -0.00214 | 0.010653 | 0.002285 | 31.39581 |
| 12 | rs11621908 | C | T | 0.024095 | 0.017706 | 0.019256 | 0.004163 | 33.50031 |
| 13 | rs11643715 | C | G | -0.0139 | -0.00337 | 0.012536 | 0.002497 | 30.97606 |
| 14 | rs11885663 | C | T | -0.01622 | -0.00674 | 0.012289 | 0.002618 | 38.37049 |
| 15 | rs12246842 | A | G | 0.013395 | 0.025991 | 0.011913 | 0.002274 | 34.68986 |
| 16 | rs12567114 | G | A | -0.01483 | -0.02621 | 0.013015 | 0.00254 | 34.08421 |
| 17 | rs12607679 | T | C | 0.020139 | -0.01441 | 0.012522 | 0.002593 | 60.32696 |
| 18 | rs12791153 | A | T | -0.02355 | -0.00138 | 0.022621 | 0.004217 | 31.18352 |
| 19 | rs13088093 | T | G | -0.01627 | -0.01593 | 0.012103 | 0.002402 | 45.87964 |
| 20 | rs13109404 | T | G | 0.031204 | 0.0544 | 0.02263 | 0.004408 | 50.1033 |
| 21 | rs151014368 | G | A | -0.01609 | 0.022107 | 0.014653 | 0.00282 | 32.57413 |
| 22 | rs1517572 | A | C | -0.01464 | -0.02261 | 0.011293 | 0.002295 | 40.72834 |
| 23 | rs1553132 | A | G | -0.01451 | 0.002374 | 0.012235 | 0.002584 | 31.50651 |
| 24 | rs17427571 | A | G | 0.013826 | 0.006106 | 0.011393 | 0.002435 | 32.22603 |
| 25 | rs174560 | T | C | -0.01358 | 0.008726 | 0.011629 | 0.002437 | 31.03588 |
| 26 | rs17732997 | C | G | 0.012935 | -0.00511 | 0.011259 | 0.002288 | 31.95298 |
| 27 | rs1776776 | T | C | 0.019963 | -0.00376 | 0.014735 | 0.003411 | 34.258 |
| 28 | rs1939455 | G | T | 0.020425 | 0.02215 | 0.016979 | 0.003561 | 32.89645 |
| 29 | rs205024 | C | T | -0.01383 | 0.007633 | 0.010783 | 0.002327 | 35.29926 |
| 30 | rs2072727 | T | C | 0.013243 | -0.01377 | 0.011046 | 0.002285 | 33.58495 |
| 31 | rs2079070 | C | G | 0.017548 | -0.00476 | 0.012963 | 0.002566 | 46.75075 |
| 32 | rs2192528 | A | G | 0.013369 | 0.00049 | 0.011005 | 0.002269 | 34.70826 |
| 33 | rs2231265 | A | G | -0.01496 | 0.012378 | 0.013055 | 0.002699 | 30.69816 |
| 34 | rs269054 | T | A | -0.01364 | -0.0049 | 0.011477 | 0.002293 | 35.39287 |
| 35 | rs2717076 | C | T | -0.01841 | 0.010409 | 0.011254 | 0.002341 | 61.85448 |
| 36 | rs3027234 | C | T | 0.015154 | 0.01154 | 0.013453 | 0.002706 | 31.35351 |
| 37 | rs3095508 | C | A | 0.015352 | 0.003371 | 0.010849 | 0.002304 | 44.38275 |
| 38 | rs34354917 | C | A | 0.013746 | 0.009932 | 0.012707 | 0.002501 | 30.21458 |
| 39 | rs34556183 | A | G | 0.016923 | 0.015412 | 0.012345 | 0.002523 | 44.98117 |
| 40 | rs34731055 | C | T | -0.01946 | 0.004609 | 0.014522 | 0.002948 | 43.58218 |
| 41 | rs35531607 | T | C | -0.01284 | 0.012901 | 0.010638 | 0.002273 | 31.91749 |
| 42 | rs35662245 | T | A | -0.0146 | -0.00699 | 0.011025 | 0.002393 | 37.21896 |
| 43 | rs365663 | A | G | 0.014629 | 0.020748 | 0.011279 | 0.002279 | 41.22024 |
| 44 | rs374153 | C | T | 0.017612 | -0.00083 | 0.014611 | 0.003103 | 32.21265 |
| 45 | rs3751046 | A | G | -0.01941 | -0.00361 | 0.014082 | 0.003208 | 36.61537 |
| 46 | rs4592416 | A | G | -0.01468 | -0.0022 | 0.010728 | 0.00227 | 41.83385 |
| 47 | rs465700 | C | G | 0.022515 | 0.017231 | 0.017971 | 0.003674 | 37.56191 |
| 48 | rs4767550 | A | G | -0.0143 | -0.00469 | 0.011618 | 0.00231 | 38.33581 |
| 49 | rs4841498 | C | T | 0.013995 | 0.00471 | 0.011589 | 0.002269 | 38.03798 |
| 50 | rs55658675 | C | T | 0.013142 | -0.02506 | 0.011564 | 0.002369 | 30.77436 |
| 51 | rs56372231 | C | T | -0.01694 | -0.01124 | 0.011113 | 0.0024 | 49.8509 |
| 52 | rs61796569 | C | T | -0.01544 | -0.01791 | 0.012756 | 0.002564 | 36.27013 |
| 53 | rs61985058 | C | T | -0.01859 | -0.0075 | 0.016826 | 0.003229 | 33.16035 |
| 54 | rs62120041 | T | C | 0.026111 | -0.02909 | 0.023355 | 0.004575 | 32.5747 |
| 55 | rs6575005 | T | C | 0.015564 | -0.00821 | 0.011868 | 0.002642 | 34.7098 |
| 56 | rs7198661 | T | C | 0.013383 | 0.014985 | 0.011047 | 0.002274 | 34.62043 |
| 57 | rs73219758 | G | A | 0.016401 | -0.00031 | 0.012426 | 0.002495 | 43.20357 |
| 58 | rs75539574 | A | C | -0.03625 | -0.00254 | 0.021565 | 0.004065 | 79.50689 |
| 59 | rs7556815 | G | A | -0.04072 | -0.00462 | 0.012969 | 0.00274 | 220.8736 |
| 60 | rs7644809 | T | C | 0.013062 | -0.00053 | 0.011533 | 0.002301 | 32.21155 |
| 61 | rs7806045 | T | C | 0.014792 | 0.021996 | 0.012515 | 0.002626 | 31.73342 |
| 62 | rs7915425 | T | C | 0.019064 | 0.005261 | 0.014127 | 0.00299 | 40.66265 |
| 63 | rs8038326 | A | G | 0.01592 | -0.00203 | 0.012827 | 0.002541 | 39.2646 |
| 64 | rs915416 | C | G | 0.019259 | -0.00235 | 0.011558 | 0.002495 | 59.60462 |
| 65 | rs9345234 | A | C | -0.01301 | -0.0138 | 0.011104 | 0.002299 | 32.0344 |
| 66 | rs9382445 | T | C | 0.014536 | 0.001834 | 0.010929 | 0.002334 | 38.79149 |
| 67 | rs9937053 | G | A | 0.016935 | 0.001698 | 0.010656 | 0.00229 | 54.66806 |

Table 7. SNPs used for univariable MR analysis estimating the effect of sleep duration on COVID-19 susceptibility, EA=effect_allele, OA=other_allele

|  | SNP | EA | OA | beta.exposure | beta.outcome | se.outcome | se.exposure | F value |
| --- | --- | --- | --- | --- | --- | --- | --- | --- |
| 1 | rs10173260 | T | C | -0.01284 | 0.009019 | 0.00423 | 0.002313 | 30.79496 |
| 2 | rs10421649 | T | A | -0.0133 | -0.00315 | 0.004432 | 0.002295 | 33.58292 |
| 3 | rs10483350 | A | G | -0.01737 | -0.01093 | 0.005304 | 0.002868 | 36.68702 |
| 4 | rs10761674 | C | T | 0.012333 | -0.00935 | 0.004056 | 0.002266 | 29.6241 |
| 5 | rs1079727 | T | C | -0.01829 | -0.00479 | 0.005581 | 0.003103 | 34.74056 |
| 6 | rs10973207 | G | T | -0.02043 | 0.007342 | 0.005661 | 0.003124 | 42.78555 |
| 7 | rs11039544 | G | A | 0.018389 | -0.00076 | 0.005786 | 0.003074 | 35.7947 |
| 8 | rs11190970 | G | A | 0.015379 | 0.001255 | 0.00534 | 0.002823 | 29.6742 |
| 9 | rs112230981 | A | G | 0.031528 | -0.00208 | 0.011093 | 0.005228 | 36.36885 |
| 10 | rs113113059 | T | C | 0.016141 | -0.00758 | 0.004964 | 0.002737 | 34.76868 |
| 11 | rs11567976 | C | T | -0.0128 | 0.004515 | 0.004147 | 0.002285 | 31.39581 |
| 12 | rs11621908 | C | T | 0.024095 | 0.007662 | 0.007458 | 0.004163 | 33.50031 |
| 13 | rs11643715 | C | G | -0.0139 | -0.00398 | 0.004643 | 0.002497 | 30.97606 |
| 14 | rs11885663 | C | T | -0.01622 | -0.00522 | 0.004804 | 0.002618 | 38.37049 |
| 15 | rs12246842 | A | G | 0.013395 | -0.00052 | 0.00429 | 0.002274 | 34.68986 |
| 16 | rs12567114 | G | A | -0.01483 | 0.00578 | 0.004758 | 0.00254 | 34.08421 |
| 17 | rs12607679 | T | C | 0.020139 | 0.001731 | 0.004806 | 0.002593 | 60.32696 |
| 18 | rs12791153 | A | T | -0.02355 | 0.011645 | 0.008082 | 0.004217 | 31.18352 |
| 19 | rs13088093 | T | G | -0.01627 | 0.00205 | 0.004456 | 0.002402 | 45.87964 |
| 20 | rs13109404 | T | G | 0.031204 | -0.04095 | 0.008698 | 0.004408 | 50.1033 |
| 21 | rs151014368 | G | A | -0.01609 | -0.00044 | 0.005318 | 0.00282 | 32.57413 |
| 22 | rs1517572 | A | C | -0.01464 | -0.00112 | 0.004245 | 0.002295 | 40.72834 |
| 23 | rs1553132 | A | G | -0.01451 | -0.00119 | 0.00482 | 0.002584 | 31.50651 |
| 24 | rs17427571 | A | G | 0.013826 | 0.000282 | 0.004431 | 0.002435 | 32.22603 |
| 25 | rs174560 | T | C | -0.01358 | 0.00232 | 0.004465 | 0.002437 | 31.03588 |
| 26 | rs17732997 | C | G | 0.012935 | 0.004394 | 0.004244 | 0.002288 | 31.95298 |
| 27 | rs1776776 | T | C | 0.019963 | 0.002126 | 0.005999 | 0.003411 | 34.258 |
| 28 | rs1939455 | G | T | 0.020425 | 0.00592 | 0.006509 | 0.003561 | 32.89645 |
| 29 | rs205024 | C | T | -0.01383 | -0.00233 | 0.004224 | 0.002327 | 35.29926 |
| 30 | rs2072727 | T | C | 0.013243 | 0.003283 | 0.004167 | 0.002285 | 33.58495 |
| 31 | rs2079070 | C | G | 0.017548 | -0.00248 | 0.004859 | 0.002566 | 46.75075 |
| 32 | rs2192528 | A | G | 0.013369 | -0.00132 | 0.004207 | 0.002269 | 34.70826 |
| 33 | rs2231265 | A | G | -0.01496 | -0.00701 | 0.00498 | 0.002699 | 30.69816 |
| 34 | rs269054 | T | A | -0.01364 | -0.00218 | 0.004223 | 0.002293 | 35.39287 |
| 35 | rs2717076 | C | T | -0.01841 | 0.001594 | 0.004296 | 0.002341 | 61.85448 |
| 36 | rs3027234 | C | T | 0.015154 | -0.00406 | 0.005127 | 0.002706 | 31.35351 |
| 37 | rs3095508 | C | A | 0.015352 | 0.00602 | 0.004215 | 0.002304 | 44.38275 |
| 38 | rs34354917 | C | A | 0.013746 | 0.004345 | 0.005019 | 0.002501 | 30.21458 |
| 39 | rs34556183 | A | G | 0.016923 | 0.00289 | 0.0051 | 0.002523 | 44.98117 |
| 40 | rs34731055 | C | T | -0.01946 | 0.002603 | 0.005566 | 0.002948 | 43.58218 |
| 41 | rs35531607 | T | C | -0.01284 | -0.00127 | 0.004117 | 0.002273 | 31.91749 |
| 42 | rs35662245 | T | A | -0.0146 | 0.001428 | 0.004334 | 0.002393 | 37.21896 |
| 43 | rs365663 | A | G | 0.014629 | -0.00388 | 0.004213 | 0.002279 | 41.22024 |
| 44 | rs374153 | C | T | 0.017612 | -0.00304 | 0.005643 | 0.003103 | 32.21265 |
| 45 | rs3751046 | A | G | -0.01941 | 0.010755 | 0.005712 | 0.003208 | 36.61537 |
| 46 | rs4592416 | A | G | -0.01468 | 0.008028 | 0.004141 | 0.00227 | 41.83385 |
| 47 | rs465700 | C | G | 0.022515 | 0.003486 | 0.006612 | 0.003674 | 37.56191 |
| 48 | rs4767550 | A | G | -0.0143 | -0.0009 | 0.004278 | 0.00231 | 38.33581 |
| 49 | rs4841498 | C | T | 0.013995 | -0.00377 | 0.004648 | 0.002269 | 38.03798 |
| 50 | rs55658675 | C | T | 0.013142 | 0.002826 | 0.004406 | 0.002369 | 30.77436 |
| 51 | rs56372231 | C | T | -0.01694 | 0.006514 | 0.004391 | 0.0024 | 49.8509 |
| 52 | rs61796569 | C | T | -0.01544 | 0.006847 | 0.004787 | 0.002564 | 36.27013 |
| 53 | rs61985058 | C | T | -0.01859 | -0.00359 | 0.00626 | 0.003229 | 33.16035 |
| 54 | rs62120041 | T | C | 0.026111 | 0.001156 | 0.008836 | 0.004575 | 32.5747 |
| 55 | rs6575005 | T | C | 0.015564 | -0.00374 | 0.004731 | 0.002642 | 34.7098 |
| 56 | rs7198661 | T | C | 0.013383 | -0.0076 | 0.004247 | 0.002274 | 34.62043 |
| 57 | rs73219758 | G | A | 0.016401 | -0.00628 | 0.004739 | 0.002495 | 43.20357 |
| 58 | rs75539574 | A | C | -0.03625 | 0.001957 | 0.007966 | 0.004065 | 79.50689 |
| 59 | rs7556815 | G | A | -0.04072 | 0.005379 | 0.005028 | 0.00274 | 220.8736 |
| 60 | rs7644809 | T | C | 0.013062 | 0.003648 | 0.004232 | 0.002301 | 32.21155 |
| 61 | rs7806045 | T | C | 0.014792 | 0.006396 | 0.004765 | 0.002626 | 31.73342 |
| 62 | rs7915425 | T | C | 0.019064 | -0.00917 | 0.005487 | 0.00299 | 40.66265 |
| 63 | rs8038326 | A | G | 0.01592 | -0.00194 | 0.004717 | 0.002541 | 39.2646 |
| 64 | rs915416 | C | G | 0.019259 | 0.003709 | 0.00451 | 0.002495 | 59.60462 |
| 65 | rs9345234 | A | C | -0.01301 | 0.000877 | 0.004189 | 0.002299 | 32.0344 |
| 66 | rs9382445 | T | C | 0.014536 | 0.0002 | 0.004262 | 0.002334 | 38.79149 |
| 67 | rs9937053 | G | A | 0.016935 | 0.000257 | 0.004178 | 0.00229 | 54.66806 |

Table 8. SNPs used for univariable MR analysis estimating the effect of COVID-19 susceptibility on insomnia, EA=effect_allele, OA=other_allele

|  | SNP | EA | OA | beta.exposure | beta.outcome | se.outcome | se.exposure | F value |
| --- | --- | --- | --- | --- | --- | --- | --- | --- |
| 1 | rs148063273 | C | T | 0.12994 | 0.012583 | 0.006953 | 0.022564 | 33.16298 |
| 2 | rs2109069 | G | A | 0.033064 | 0.002307 | 0.001481 | 0.004684 | 49.82628 |
| 3 | rs34288077 | A | G | 0.077427 | 0.001071 | 0.002733 | 0.007723 | 100.5212 |
| 4 | rs4342086 | G | A | -0.04049 | 0.00108 | 0.001451 | 0.004424 | 83.75865 |
| 5 | rs4801778 | G | T | -0.03515 | -0.0014 | 0.001772 | 0.00549 | 41.0003 |
| 6 | rs505922 | C | T | -0.07428 | 6.42E-05 | 0.001487 | 0.00427 | 302.6661 |
| 7 | rs73062389 | G | A | 0.16225 | -7.66E-05 | 0.002912 | 0.00949 | 292.3363 |

Table 9. SNPs used for univariable MR analysis estimating the effect of COVID-19 susceptibility on sleep duration, EA=effect_allele, OA=other_allele

|  | SNP | EA | OA | beta.exposure | beta.outcome | se.outcome | se.exposure | F value |
| --- | --- | --- | --- | --- | --- | --- | --- | --- |
| 1 | rs148063273 | C | T | 0.12994 | -0.00068 | 0.01141 | 0.022564 | 33.16298 |
| 2 | rs2109069 | G | A | 0.033064 | -0.00191 | 0.002421 | 0.004684 | 49.82628 |
| 3 | rs34288077 | A | G | 0.077427 | 0.005598 | 0.004471 | 0.007723 | 100.5212 |
| 4 | rs4342086 | G | A | -0.04049 | -0.00275 | 0.002374 | 0.004424 | 83.75865 |
| 5 | rs4801778 | G | T | -0.03515 | -0.00044 | 0.002899 | 0.00549 | 41.0003 |
| 6 | rs505922 | C | T | -0.07428 | -0.00874 | 0.002436 | 0.00427 | 302.6661 |
| 7 | rs73062389 | G | A | 0.16225 | 0.002662 | 0.004774 | 0.00949 | 292.3363 |
