# Supplementary material for: Insomnia and sleep duration on COVID-19 susceptibility and hospitalization: A Mendelian randomization study
Source: Front Public Health. 2022 Sep 30;10:995664. doi: 10.3389/fpubh.2022.995664 (PMC9561394; doi:10.3389/fpubh.2022.995664)
Supplement: Supplementary file 2 [file Data_Sheet_2.docx]

To test the reverse causality, we performed reverse MR analysis. Reverse MR result showed only a causal association between COVID-19 susceptibility and sleep duration (Beta=0.05, 95%CI: 0.01, 0.09; *p* =0.02)

Table 1. The results of reverse MR

| Exposure | Outcome | Methods | nSNPs | OR/Beta  (95%CI) | p |
| --- | --- | --- | --- | --- | --- |
| COVID-19 Susceptibility | Insomnia | IVW | 7 | 1.01(0.98,1.03) | 0.46 |
|  |  | MR-Egger | 7 | 1.00(0.62,1.59) | 0.92 |
|  |  | Egger-intercept |  | 0.00082 | 0.62 |
|  |  | WME | 7 | 1.00(0.97,1.03) | 0.97 |
| COVID-19 hospitalization | Insomnia | IVW | 14 | 1.00(0.99,1.01) | 0.46 |
|  |  | MR-Egger | 14 | 1.01(0.99,1.04) | 0.35 |
|  |  | Egger-intercept |  | -0.0020 | 0.16 |
|  |  | WME | 14 | 1.00(0.99,1.01) | 0.75 |
| COVID-19 Susceptibility | Sleep duration | IVW | 7 | 0.05(0.01,0.09) | 0.02 |
|  |  | MR-Egger | 7 | 0.05(-0.04,0.14) | 0.30 |
|  |  | Egger-intercept |  | -0.0002 | 0.95 |
|  |  | WME | 7 | 0.05(0.00,0.10) | 0.05 |
| COVID-19 hospitalization | Sleep duration | IVW | 14 | 0.013(0.01,0.04) | 0.34 |
|  |  | MR-Egger | 14 | 0.02(-0.04,0.08) | 0.54 |
|  |  | Egger-intercept |  | -0.0008 | 0.82 |
|  |  | WME | 14 | 0.02(-0.004,0.03) | 0.12 |

In order to verify whether sleep duration is a collider factor on the pathway from insomnia to COVID-19 Susceptibility, we performed a univariate MR analysis of insomnia on sleep duration. Result showed that insomnia had an impact on sleep duration (Beta=-0.71, 95%CI: -0.91, -0.50; *p*$<$0.001)

Table 2. The effect of insomnia on sleep duration

| Exposure | Outcome | Methods | nSNPs | Beta(95%CI) | P-value |
| --- | --- | --- | --- | --- | --- |
| Insomnia | Sleep duration | IVW | 41 | -0.71(-0.91,-0.50） | 8.61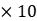^-12^ |
|  |  | MR-Egger | 41 | -0.51(-1.23,0.20) | 0.168 |
|  |  | Egger-intercept | 41 | -1.38 | 0.996 |
|  |  | WME | 41 | -0.46(-0.60,-0.31) | 8.36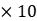^-10^ |
